# Prevalence of Anaerobic Bacteria in Surgical Site Infections in a Tertiary Care Hospital

**DOI:** 10.7759/cureus.107959

**Published:** 2026-04-29

**Authors:** Ratnadeep Kedia, S Sivasankari, S Senthamarai, Akila K, Subha Vajjiravel Jagannath, Anitha Srinivasagalu, Sekhar Ambuja

**Affiliations:** 1 Department of Microbiology, Meenakshi Medical College Hospital and Research Institute, Meenakshi Academy of Higher Education and Research, Kanchipuram, IND

**Keywords:** anaerobic bacteria, anaerobic culture, bacteroides spp, polymicrobial infection, postoperative wound infection, surgical site infection

## Abstract

Background

Surgical site infections (SSIs) are important postoperative complications associated with prolonged hospitalization, increased antimicrobial exposure and higher healthcare costs. Anaerobic bacteria are recognized contributors to SSIs but are often underdetected in routine laboratory practice due to challenges in specimen collection, transport and culture. This study aimed to determine the prevalence of anaerobic bacteria in clinically suspected SSIs and to describe the distribution of anaerobic isolates in monomicrobial and polymicrobial infections.

Methods

A prospective cross-sectional study was conducted in the Department of Microbiology at a tertiary care teaching hospital in Kanchipuram, India, from November 2024 to September 2025. A total of 72 postoperative patients with clinical suspicion of SSI were included. Appropriate wound specimens were processed for aerobic and anaerobic culture using standard microbiological methods. Anaerobic isolates were identified to the genus level by phenotypic disc-based and biochemical methods. Data were analyzed using IBM SPSS Statistics for Windows Version 29.0 and summarized using descriptive statistics.

Results

Anaerobic organisms were recovered from 22/72 specimens (30.5%). Among anaerobic culture-positive cases, polymicrobial infection was more common than monomicrobial infection, occurring in 16/22 (72.7%) and 6/22 (27.3%) cases, respectively. *Bacteroides* spp. were the most frequent isolates, accounting for 11/22 (50.0%), followed by *Prevotella* spp. in 5/22 (22.7%) cases. Anaerobic culture-positive cases were most common in patients aged 41-60 years (9/22, 40.9%), in men (14/22, 63.6%), and in gastrointestinal surgery cases (10/22, 45.5%).

Conclusions

Anaerobic bacteria constituted a substantial component of SSI microbiology, particularly in gastrointestinal and polymicrobial postoperative wound infections. These findings support the routine consideration of anaerobic culture in clinically suspected deep or contaminated SSIs to improve etiological diagnosis and guide antimicrobial management.

## Introduction

Surgical site infection (SSI) remains one of the most consequential complications of operative care because it prolongs recovery, increases antimicrobial exposure, escalates cost and may convert an otherwise controlled surgical event into a complex infectious syndrome. Across the broader literature, the burden of SSIs continues to be substantial, although its reported frequency varies according to procedure-type wound class, patient risk profile and local surveillance intensity. Reviews by Nichols [1], Rezaei et al. [2] and Gouda [3] emphasize that SSI is not a single microbiological entity but a heterogeneous postoperative complication shaped by tissue disruption, endogenous flora, operative contamination and perioperative host factors. This distinction matters because the microbiology of SSI changes with the surgical field. Clean procedures are classically dominated by skin commensals, especially *Staphylococcus aureus,* whereas clean, contaminated and dirty operations more often yield mixed endogenous flora, including enteric facultative organisms and obligate anaerobes [1,2,4]. Yet, in routine practice, the diagnostic lens often remains disproportionately aerobic. As a result, anaerobic pathogens are frequently underdetected not because they are unimportant, but because their recovery depends on proper specimen selection, rapid transport, suitable anaerobic media and deliberate laboratory processing. That diagnostic gap has direct therapeutic implications since missed anaerobic involvement can distort empirical treatment and obscure the true ecological complexity of postoperative wound infection.

The importance of anaerobes in surgical infections has been recognized for decades but is still not consistently reflected in routine microbiological workup. Classical studies demonstrated that postoperative wounds, particularly those related to abdominal or deep tissue surgery often harbor mixed aerobic anaerobic flora rather than isolated single pathogens. Sanderson et al. [5] showed striking recovery of anaerobes from postoperative wound infections, especially after abdominal operations with *Bacteroides* species predominating. Likewise, Brook [6] reported that even postthoracotomy sternal wound infection was not exclusively aerobic or staphylococcal because anaerobes and mixed flora formed a meaningful subset of cases. Brook and Frazier [7] further reinforced this point in spinal fusion SSI where nearly half of infections showed mixed aerobic and anaerobic growth and the *Bacteroides fragilis* group emerged as a major anaerobic component. These observations are microbiologically plausible because devitalized tissue, impaired perfusion, hematoma, foreign material and reduced oxidation-reduction potential create a favorable niche for anaerobic survival, while facultative organisms may further facilitate anaerobic growth through oxygen consumption and synergistic tissue damage [4,8]. The concept of polymicrobial synergy, therefore, becomes central rather than incidental in understanding many postoperative wound infections.

More recent work has extended this older microbiological insight into contemporary hospital settings. Nema et al. [9] showed that obligate anaerobes were recovered from pyogenic infections both alone and in mixed culture, underscoring that anaerobic disease is neither rare nor confined to exotic deep abscesses. Padmaja Ananth-Shenoy et al. [10] similarly found that anaerobic infections in surgical patients were predominantly mixed and were led by anaerobic Gram-negative bacilli, especially the *B. fragilis* group and *Prevotella* species. Akhi et al. [11] also documented significant anaerobic participation in SSI and highlighted the therapeutic importance of culture-directed management in an era of variable resistance. At the same time, the literature shows striking interstudy variation in anaerobic yield. Chukwuma et al. [12] identified anaerobic SSI infrequently, whereas Behera et al. [13] demonstrated that even conventionally culture-negative SSI may conceal anaerobes and other overlooked organisms when molecular tools are used. This variation suggests that the apparent burden of anaerobic SSI depends not only on patient and surgical factors but also on how actively laboratories seek these organisms. In abdominal and gastrointestinal surgery, where endogenous bowel flora drive contamination risk, the relevance of anaerobic bacteriology becomes even greater [14,15].

Against this background, the present study was undertaken to examine the prevalence of anaerobic bacteria in clinically SSIs and to characterize the distribution of anaerobic isolates and their occurrence in monomicrobial and polymicrobial infections in a tertiary care hospital.

## Materials and methods

A prospective cross-sectional study was conducted in the Department of Microbiology at Meenakshi Medical College Hospital and Research Institute, Kanchipuram, over an 11-month period from November 2024 to September 2025. Institutional Ethics Committee approval was obtained before commencement of the study (MMCH&RI IEC/PG/01/Nov/24).

Study population

The study included 72 postoperative patients with clinical suspicion of SSI from various surgical units of the hospital. Patients of all age groups who underwent clean as well as unclean surgical procedures were eligible for inclusion. Cases were enrolled after the development of clinical evidence suggestive of postoperative wound infection. A total of 12 specimens were excluded because they were received in open or leaking containers or were suboptimal samples, such as wound swabs.

Clinical data collection

Demographic and clinical details were collected using a structured pro forma and supplemented from patient medical records. The recorded variables included age, sex, type of surgery, ward of admission, wound classification, duration of surgery, prior antibiotic exposure, associated comorbidities including diabetes mellitus, and relevant postoperative clinical course.

Specimen collection and transport

Appropriate wound specimens, including pus, pus aspirate, necrotic material, and drain fluid, were collected aseptically in sterile wide-mouthed containers. Simultaneously, specimens were inoculated into Robertson's cooked meat medium for anaerobic processing. To preserve anaerobic viability, all specimens were transported to the microbiology laboratory and processed within 30 minutes of collection. No delays beyond 30 minutes occurred.

Microbiological processing

All specimens were subjected to direct Gram staining and processed for both aerobic and anaerobic culture using standard microbiological techniques. For anaerobic isolation, specimens were inoculated onto 5% sheep blood agar, neomycin blood agar, and Brucella blood agar with a metronidazole disc for preliminary screening. Robertson's cooked meat broth was incubated for up to seven days. Subculture onto 5% sheep blood agar was performed whenever broth examination or Gram stain demonstrated additional morphotypes suggestive of anaerobic growth. Culture media showing no growth after seven days of incubation were discarded.

For aerobic culture, specimens were inoculated onto 5% sheep blood agar and MacConkey agar and incubated under standard aerobic conditions. Aerobic isolates were identified by conventional microbiological methods.

Identification of anaerobic isolates

Presumptive identification of anaerobic isolates was performed by the AnIdent special potency disc method using vancomycin 5 µg, kanamycin 1000 µg, and colistin 10 µg discs. Further characterization was based on colony morphology, Gram stain appearance, aerotolerance, and standard biochemical reactions, including catalase production, nitrate reduction, growth in 20% bile, esculin hydrolysis, lipase production, lecithinase production, susceptibility to sodium polyanethol sulfonate, urease activity, and sugar fermentation in Viande-Levure broth. Anaerobic isolates were identified to the genus level on the basis of phenotypic and biochemical characteristics.

Definitions used in analysis

Anaerobic culture positivity was defined as recovery of one or more anaerobic organisms from the postoperative wound specimen. Cases yielding only one anaerobic organism were categorized as monomicrobial anaerobic infection, whereas cases yielding anaerobic organisms in combination with one or more aerobic bacteria were categorized as polymicrobial infection.

Statistical analysis

Data were entered and analyzed using IBM SPSS Statistics for Windows, Version 29.0 (IBM Corp, Armonk, NY). Categorical variables were summarized as frequencies and percentages. The anaerobic culture positivity rate was calculated using the total number of postoperative wound specimens processed as the denominator. The distribution of anaerobic isolates and the demographic and surgical characteristics of anaerobic culture-positive cases were described using descriptive statistics only. No inferential statistical tests were applied; therefore, p-values, significance thresholds, and test statistics were not generated. Accordingly, subgroup distributions were interpreted as descriptive patterns only and not as statistically significant differences.

## Results

A total of 72 postoperative wound specimens from patients with clinical suspicion of SSI were processed for anaerobic culture. Anaerobic organisms were recovered from 22/72 (30.6%) specimens, whereas 50/72 (69.4%) specimens showed no anaerobic growth. Among the 22 anaerobic culture-positive cases, polymicrobial infection predominated in 16/22 (72.7%) cases, whereas monomicrobial anaerobic infection was identified in 6/22 (27.3%) cases. In polymicrobial infections, anaerobes were recovered together with aerobic organisms, including *Escherichia coli*, *Klebsiella* spp., *Pseudomonas aeruginosa*, *Proteus* spp., and *Enterococcus* spp. This pattern indicates that anaerobic SSI in the present series was more often part of a mixed-wound microbiota rather than an isolated microbiological event. Such predominance of mixed aerobic-anaerobic infection is in keeping with published literature on postoperative and deep wound infections, where polymicrobial synergy has been shown to be a defining microbiological feature of anaerobic surgical infections (Table 1).

**Table 1:** Distribution of anaerobic isolates recovered from surgical site infections Data are presented as n (%). Percentages were calculated using the total number of anaerobic isolates recovered (N=22). The frequencies of less common isolates were low and are presented for descriptive microbiological completeness only. No inferential statistical comparison was performed.

Anaerobic organism	Number of isolates	Percentage (%)
*Bacteroides* spp.	11	50.0
*Prevotella* spp.	5	22.7
*Porphyromonas* spp.	2	9.1
*Fusobacterium* spp.	2	9.1
*Peptostreptococcus* spp.	2	9.1
Total	22	100.0

Among the 22 anaerobic isolates, *Bacteroides* spp. were the predominant organisms, accounting for half of all isolates (50.0%) followed by *Prevotella* spp. at 22.7%. *Porphyromonas* spp., *Fusobacterium* spp. and *Peptostreptococcus* spp. each contributed 9.1% of the isolates. Overall, the isolate profile showed clear predominance of Gram-negative anaerobic bacilli, particularly *Bacteroides* and *Prevotella*, over anaerobic Gram-positive cocci. This distribution is microbiologically plausible and aligns with earlier reports in surgical and wound infections in which the *B. fragilis* group and related pigmented Gram-negative anaerobes were frequently dominant, especially in infections linked to gastrointestinal or mixed endogenous flora (Table 2).

**Table 2:** Age and sex distribution of anaerobic culture-positive cases Data are presented as n (%). Percentages were calculated using the total number of anaerobic culture-positive cases (n = 22). Only descriptive statistics were applied.

Variable	Category	Number of cases (n=22)	Percentage (%)
Age group (years)	<20	2	9.1
	21-40	5	22.7
	41-60	9	40.9
	>60	6	27.3
Sex	Male	14	63.6
	Female	8	36.4

Age-wise distribution showed that anaerobic culture-positive SSI was most frequent in the 41-60-year age group (9/22 cases, 40.9%) followed by patients older than 60 years (27.3%). Younger age groups contributed comparatively fewer cases. Sex distribution showed a male predominance with 14/22 cases (63.6%) occurring in men. Although no inferential comparison was performed, this pattern suggests that anaerobic SSI in the present cohort clustered more often in middle-aged to older adults and in men, likely reflecting the underlying surgical case mix and exposure to major abdominal and general surgical procedures rather than a sex-specific microbiological predisposition (Table 3).

**Table 3:** Distribution of anaerobic culture-positive cases according to type of surgery and ward Data are presented as n (%). Percentages were calculated using the total number of anaerobic culture-positive cases (n=22). Only descriptive statistics were applied.

Category	Type	Number of cases	Percentage (%)
Type of surgery	Gastrointestinal surgery	10	45.5
	Orthopaedic surgery	4	18.2
	Obstetrics and gynaecology surgery	3	13.6
	Urological surgery	3	13.6
	Other surgical procedures	2	9.1
Ward	General Surgery	11	50.0
	Orthopaedics	4	18.2
	Obstetrics and Gynaecology	3	13.6
	Urology	2	9.1
	Other surgical wards	2	9.1

## Discussion

Key findings

The present study shows that anaerobic bacteria formed a clinically meaningful component of SSI rather than an incidental microbiological finding. Anaerobic organisms were recovered from 22 of 72 clinically suspected postoperative wound specimens, giving an anaerobic culture positivity rate of 30.5%. Among these anaerobic culture-positive cases, polymicrobial infection predominated at 72.7%, while monomicrobial anaerobic infection accounted for only 27.3% of the cases. The isolate profile was led by *Bacteroides* spp. (50.0%) followed by *Prevotella* spp. (22.7%), with smaller contributions from *Porphyromonas*, *Fusobacterium*, and *Peptostreptococcus*. Anaerobic culture-positive cases clustered in the 41-60-year age group, showed male predominance, and were most frequently associated with gastrointestinal surgery and general surgery wards. Taken together, these findings suggest that in this setting, anaerobic SSI is chiefly a mixed endogenous postoperative infection, especially in operations involving bowel-associated flora and deeper tissue disruption.

Agreement with the existing literature

These observations are strongly concordant with classical and contemporary literature. The predominance of mixed aerobic-anaerobic infection in the present series mirrors the central microbiological principle described by Bowler et al. [4] that wound infection is often polymicrobial and shaped by synergistic interactions rather than by a single pathogen alone. It also aligns closely with Brook and Frazier [7], who documented mixed growth in 48% of spinal fusion SSI, and with Padmaja Ananth-Shenoy et al. [10], who found mixed aerobic-anaerobic infection in 71.9% of anaerobic surgical infections. Likewise, the dominance of *Bacteroides* and other Gram-negative anaerobic bacilli in the current study is consistent with Sanderson et al. [5], who showed marked recovery of *Bacteroides* from postoperative wound sepsis, particularly after abdominal procedures, and with Akhi et al. [11] and Angrup et al. [16], who also identified *Bacteroides* as a major anaerobic pathogen in surgical and mixed anaerobic infections. The concentration of cases in gastrointestinal surgery is biologically expected, because abdominal and bowel-related procedures expose wounds to endogenous enteric flora where obligate anaerobes naturally coexist with facultative Gram-negative bacilli such as *E. coli* and *Klebsiella* spp.

Disagreement with the literature and plausible explanations

At the same time, the anaerobic yield of 30.5% in the present study is higher than that reported in some recent studies, particularly Chukwuma et al. [12], who found anaerobic SSI in only 1.1% of all postoperative patients, and Nema et al. [9], who reported obligate anaerobes in 14.5% of pyogenic specimens. This divergence should not be interpreted as a contradiction alone; rather, it likely reflects differences in study denominator, specimen quality, case selection, and laboratory intensity. The present work examined only clinically suspected SSI cases and excluded suboptimal samples such as wound swabs, while also using prompt transport, Robertson's cooked meat medium, selective anaerobic media, and extended incubation. Such a methodology would reasonably improve anaerobic recovery. In contrast, studies with broader postoperative denominators, more superficial infections, or less optimized anaerobic workflows may underestimate the true burden. The current isolate pattern also differs from Brook [6] postthoracotomy sternal wound series, where *Peptostreptococcus* was more prominent; however, that discrepancy is plausibly explained by anatomical site, because thoracic wounds are microbiologically distinct from gastrointestinal and general surgical wounds. In the same vein, the higher anaerobic burden in the present study may also reflect local surgical case mix and a larger contribution of bowel-related contamination.

Clinical implications

The clinical message is straightforward. Routine aerobic culture alone is insufficient for a substantial subset of SSI, particularly when infection follows gastrointestinal or other contaminated procedures, when tissue is devitalized, or when clinical features suggest deeper mixed infection. Failure to seek anaerobes risks incomplete etiological diagnosis and may bias empirical therapy towards purely aerobic coverage. The present data therefore support deliberate incorporation of anaerobic culture into SSI workup, especially for aspirates, drain fluid, necrotic tissue, and deep wound material. They also reinforce the need for close clinician-microbiologist communication so that empirical therapy, source control, and later antimicrobial refinement are aligned with the expected polymicrobial ecology. Importantly, the literature also warns that antimicrobial choice should remain stewardship-based; the goal is not indiscriminate expansion of therapy but rational anaerobic coverage when the surgical context makes it microbiologically credible [8,17,18].

Limitations

This study has several limitations that should temper interpretation. First, it was a single-center study with a modest sample size and only 22 anaerobic culture-positive cases. Second, the analysis was descriptive only, so no inferential assessment of risk factors or independent predictors was possible. Third, although wound classification was recorded, the study did not perform a separate stratified analysis according to wound class; therefore, the relationship between wound contamination category and anaerobic culture positivity could not be evaluated. Fourth, anaerobic isolates were identified only to the genus level, which limits epidemiological granularity. Fifth, anaerobic antimicrobial susceptibility testing was not performed, preventing correlation between isolate pattern and therapeutic options. Sixth, molecular methods were not used; therefore, some viable but non-culturable, fastidious, or low-burden anaerobes may still have been missed, as suggested by Behera et al. [13]. Finally, outcome linkage was limited, so the study cannot determine whether anaerobic positivity altered severity, duration of hospitalization, reoperation, or treatment response.

## Conclusions

In this single-centre descriptive study, anaerobic bacteria were recovered from 30.5% of clinically suspected SSIs. Most anaerobic culture-positive cases were polymicrobial, and the predominant isolates were *Bacteroides *spp. and *Prevotella *spp., particularly among gastrointestinal and general surgical cases. These findings indicate that anaerobes may form part of the microbiological spectrum of selected postoperative wound infections, especially when the infection is deep, contaminated, or clinically suggestive of mixed flora.

However, because the study was descriptive, included a modest sample size, and identified isolates only to the genus level, the findings should be interpreted as local microbiological evidence rather than as proof of independent risk, clinical outcome impact, or treatment superiority. The study supports the selective consideration of anaerobic culture in appropriately collected SSI specimens to improve etiological documentation and assist rational antimicrobial decision-making.
